# A Multiday Evaluation of Real-Time Intramuscular EMG Usability with ANN

**DOI:** 10.3390/s20123385

**Published:** 2020-06-15

**Authors:** Asim Waris, Muhammad Zia ur Rehman, Imran Khan Niazi, Mads Jochumsen, Kevin Englehart, Winnie Jensen, Heidi Haavik, Ernest Nlandu Kamavuako

**Affiliations:** 1 Department of Biomedical Engineering and Sciences, School of Mechanical and Manufacturing Engineering (SMME), National University of Sciences and Technology (NUST), Islamabad 44000, Pakistan; asim.waris@smme.nust.edu.pk; 2 Faculty of Engineering and Applied Sciences, Riphah International University, Islamabad 46000, Pakistan; ziaur.rehman@riphah.edu.pk; 3 Center for Sensory-Motor Interaction, Department of Health Science and Technology, Aalborg University, 9220 Aalborg, Denmark; mj@hst.aau.dk (M.J.); wj@hst.aau.dk (W.J.); 4 Center of Chiropractic Research, New Zealand College of Chiropractic, Auckland 1060, New Zealand; Heidi.Haavik@nzchiro.co.nz; 5 Faculty of Health and Environmental Sciences, Health and Rehabilitation Research Institute, AUT University, Auckland 0627, New Zealand; 6 Department of Electrical and Computer Engineering, University of New Brunswick, Fredericton, NB E3B 5A3, Canada; kengleha@unb.ca; 7 Centre for Robotics Research, Department of Informatics, King’s College London, London WC2R 2LS, UK; ernest.kamavuako@kcl.ac.uk

**Keywords:** intramuscular electromyography (iEMG), prosthetic hand, pattern recognition (PR)

## Abstract

Recent developments in implantable technology, such as high-density recordings, wireless transmission of signals to a prosthetic hand, may pave the way for intramuscular electromyography (iEMG)-based myoelectric control in the future. This study aimed to investigate the real-time control performance of iEMG over time. A novel protocol was developed to quantify the robustness of the real-time performance parameters. Intramuscular wires were used to record EMG signals, which were kept inside the muscles for five consecutive days. Tests were performed on multiple days using Fitts’ law. Throughput, completion rate, path efficiency and overshoot were evaluated as performance metrics using three train/test strategies. Each train/test scheme was categorized on the basis of data quantity and the time difference between training and testing data. An artificial neural network (ANN) classifier was trained and tested on (i) data from the same day (WDT), (ii) data collected from the previous day and tested on present-day (BDT) and (iii) trained on all previous days including the present day and tested on present-day (CDT). It was found that the completion rate (91.6 ± 3.6%) of CDT was significantly better (*p* < 0.01) than BDT (74.02 ± 5.8%) and WDT (88.16 ± 3.6%). For BDT, on average, the first session of each day was significantly better (*p* < 0.01) than the second and third sessions for completion rate (77.9 ± 14.0%) and path efficiency (88.9 ± 16.9%). Subjects demonstrated the ability to achieve targets successfully with wire electrodes. Results also suggest that time variations in the iEMG signal can be catered by concatenating the data over several days. This scheme can be helpful in attaining stable and robust performance.

## 1. Introduction

The electric signals generated by the contraction of myofibers in the motor units can be recorded using either surface (noninvasive) or intramuscular (invasive) electrodes. The traditional clinical use of intramuscular electromyography (iEMG) is in the diagnoses of myopathies, myodystrophies and neuromuscular disorders. Their use in myoelectric prosthetic control is limited due to its invasiveness and instability of the implanted electrodes as well as the lack of commercially available implantable recording electrodes.

Herberts et al. were the first to implant intramuscular electrodes to estimate the contraction level of muscles by applying different loads on hand joints [1]. A few years later, Tucker and Peteleski implanted an intramuscular electrode into the functioning forearm muscle of a subject with congenital limb deficiency. The system reportedly worked well but no detail of signal processing and performance of the system was reported [2]. Stein et al. implanted four pairs of electrodes for one year and compared their performance with surface electrodes; improved direct control with greater reliability of the implanted system was reported compared to surface electrodes [3]. These studies were limited to direct myoelectric control only. Several other studies utilized intramuscular recordings for neurophysiological investigations [4,5,6,7]. The focus of these studies was to determine discharge times of individual motor units. It was also found that decomposition of surface EMG signals is more cumbersome as compared to iEMG as in surface and EMG signals are in the superimposed form. Moreover, analysis of iEMG signals showed that individual motor units can be decomposed more accurately allowing recordings from deep muscles [8,9].

Recently, a variety of implantable electrodes are being developed and tested in humans, making iEMG signals clinically viable for future myoelectric control [10,11,12,13,14,15,16]. These implantable electrodes are designed to transmit the iEMG signal wirelessly to the prosthetic hand [10]. The Myoelectric Implantable Recording Array (MIRA) can also be used in the future as such systems provide an additional option of multiple independent control sites to myoelectric control [13,15]. These wireless detection systems can be implanted by utilizing a small surface area on the amputated limb and provide up to 64 channels of EMG signal by inserting only a few wires. With the help of implanted electrodes, EMG signals can be recorded from small and deep muscles providing very localized information and the amount of information gathered can be increased significantly with these detection systems [13,15]. Furthermore, an implanted iEMG electrode may provide high interday repeatability, multiple and independent channels, stable and robust signal source that is less affected by factors such as electrode shifts, skin impedance and precipitation [16,17]. However, the selectivity of these iEMG recordings may constitute a drawback as they reflect the activity of a small number of motor units of muscle fibers located close to the detection site [17].

More recent studies using iEMG as a control signal focused on pattern recognition techniques (PR) for myoelectric control [18,19,20,21]. Smith et al. compared EMG signals recorded from the surface of the forearm sEMG and iEMG for simultaneous control of hand motions using Linear Discriminant Analysis (LDA). Higher classification accuracy was reported for iEMG compared to sEMG, parallel classifier method was used without including the 2-DOF motion class data in training [18]. Fitts’ law tests were also utilized by Kamavuako et al. to evaluate the adoption of iEMG recordings in acute settings [19]. In another study, significant differences in offline classification performances were found between sEMG and iEMG recordings using LDA [20]. Data were recorded for seven continuous days [22]. All of these studies assessed iEMG-based PR techniques in either offline or in acute settings only.

The consistent and stable performance of PR schemes is an important factor that needs to be addressed for the clinical translation of these techniques. Recently, it was depicted in the literature that between-day performances degrade over time using sEMG [21,22,23,24]. To the best of our knowledge, no iEMG-based real-time evaluation of a PR scheme has previously been conducted over multiple days. The aim of this study was to quantify the real-time subchronic usability of iEMG recordings using a Fitts’ law approach with different train/test schemes.

Training of a classifier is an important step in any PR-based technique. How this factor affects the overall performance of myoelectric control in real-time over time, if trained differently is an important question. This factor is investigated in this study by designing a novel protocol for the experiment. In a five-day experiment, intramuscular EMG recordings were used to test three different training methodologies: (i) within-day training and testing of a classifier, (ii) between-day training and testing of a classifier and (iii) combined-day training and testing of a classifier. Offline classification accuracies were also evaluated keeping the same training strategies as of real-time (i) within-day classification error (WCE), (ii) between-day classification error (BCE) and (iii) combined-day classification error (CCE).

## 2. Materials and Methods

### 2.1. Subjects

In total, five able-bodied subjects took part in the experiment. None of the subjects had any medical condition related to their muscles. The average age of the participants in the experiment was 25.4 y. Written consent was taken from all the participants in the study. The protocol of the experiments was in accordance with the Declaration of Helsinki and approved by the local ethical committee of the region of Northern Jutland (Approval Number: N-20160021).

### 2.2. Experimental Procedures

**Figure 1:**
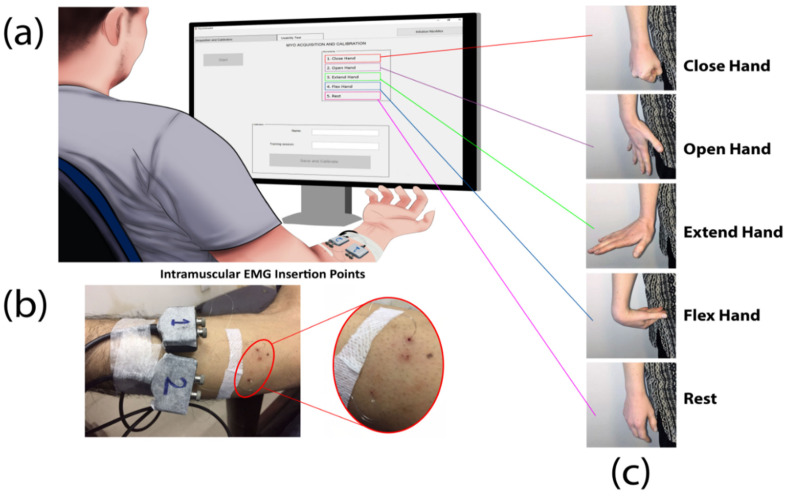
(**a**) GUI interface during the training session. Each motion was graphically displayed to the subject during training with the time-cue. (**b**) Intramuscular electrode insertion sites on the forearm of one of the subjects participated in the experiment. (**c**) Motions that each subject performed during the training session.

An EMG12 amplifier by OT Bioelectronica was used to record iEMG signals which were then passed through a bandpass filter (100–900 Hz). These filtered analog signals were amplified with a gain of 5000, at a sampling rate of 2kHz and digitized using 16 bits analog to digital converter (NI-DAQ PCI-6221). A band electrode was placed on the wrist contralateral to the dominant one as a reference. Figure 1 shows the setup for this experiment. Using three pairs of wire electrodes, iEMG was recorded from three different muscles, namely: Extensor Digitorum on Channel 1, Extensor Carpi Radialis Longus on Channel 2 and Flexor Digitorum Superficialis on Channel 3. These in vivo wire electrodes were made from 50 µm diameter Teflon-coated stainless steel. A 25-gauge sterilized needle was inserted in each muscle for each electrode. Precautionary measures against the risk of infection were thoroughly observed. Each subject’s skin was disinfected with 70% isopropyl alcohol before needle insertion. Sterile electrodes and gloves were used while handling the subjects.

The needle was inserted 10–15 mm below the muscle fascia and removed once the electrodes had been fixed inside the muscle. The insulated wires were unsheathed from the tip by about 3 mm to maximize the pickup area [19]. These electrodes were to stay in each subject’s arms for five days.

A double bandage strategy was implemented to minimize the movement of intramuscular electrodes. After the electrodes were inserted, a sterile bandage was taped on the wires leaving leeway for in vivo wire motion during extension and flexion and to allow connection to amplifiers. After each session, another bandage was placed to completely cover the wires before each subject left the room. This bandage served as a precautionary measure against electrode displacement. It was removed once the subject re-entered the room for further sessions. The bottom bandage was only removed upon the subject’s wish to withdraw or after all the sessions had been successfully completed.

### 2.3. Experimental Scheme

The experiment had two main steps: (i) data were collected to train a classifier (ii) then the models trained on different sets of collected data were tested online. For the first step, subjects were required to produce a medium level contraction from rest to motion. They were prompted by an image of a specific motion randomly generated by a customized MATLAB-based Graphical User Interface. For each motion, data were collected four times, for 6 s each time. Between each sustained contraction, a 6 s break was given. Data for four active motions (Wrist Extension, Wrist Flexion, Hand Open, Hand Close) and one rest (no motion) were collected. After each set of five motions, a break of 12 s was given.

**Figure 2:**
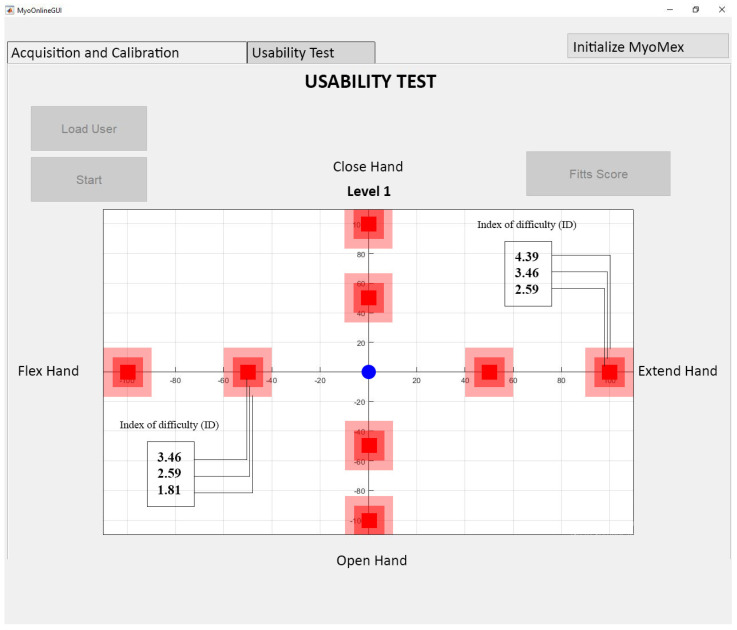
Shows the interface of GUI during the testing phase. The blue dot at the center of the XY plane corresponds to the rest position whereas red rectangles represent the target. Subjects had to achieve targets with the cursor placed at the origin in the XY plane. The target appeared randomly on any of the XX’ or YY’ axes. Each target was represented by a movement shown in Figure 1c.

For the second step, a cursor at the center of the screen was to be controlled by the hand. Figure 2 shows the GUI interface from the testing phase. There were two axes on the screen: the top YY’ represented Hand Closed while the bottom YY’ represented Hand Opened. Similarly, the left XX’ represented Hand Flexion and the right XX’ represented Hand Extension. To be considered a successful movement, the cursor had to hit the target and remain at it for one second. The entire experiment spanned five days. On each day, three types of online tests were carried out as shown in Table 1. Firstly, within-day training and testing of the artificial neural network (ANN) was denoted by the WDT (data from the same day) row shown in the table. BDT (data collected from the previous day and tested on present-day) represents the online test in which the training data of the previous day was used to test the data of the present day. Lastly, the training data of all the previous days was used to test the present-day data in CDT (trained on all previous days including the present day and tested on present-day). Each day, three sessions for testing were performed. There were four indexes of difficulty levels, thus each motion was tested 18 times in 3 sessions per day. A break of 10 minutes was given between each session. Additionally, it should be noted that, in a single session, 24 targets (4 directions × 2 distances × 4 widths) were to be reached by the subjects. Each target was unique with respect to its motion and size of the target.

### 2.4. Feature Extraction and Experimental Procedure 

A 200ms overlapping window with an increment of 50ms was used to segment the steady-state part of 4 s of the data from each 6 s recorded signal. Six features were examined, namely: Mean Absolute Value, Cardinality, Waveform Length, Zero Crossings, Willison Amplitude and Slope Sign Changes. Table 2 summarizes the description of each feature used in this study [25].

ANN was used as an offline and online training and testing classifier [26]. The network was trained with the Levenberg–Marquardt algorithm. A single hidden layer was used and after several trials, the size of the input layer was made equal to the size of the features vector (18 × 1). The offline configuration was simulated in the GUI such that it had a fixed number of neurons in the hidden layer. A profile specific to each subject was created in which the subject’s calibrated signals were stored. The trained ANN was subjected to Fitts’ law to classify the cursor-controlled hand gestures [27].

During the implementation of Fitts’ law, participants were asked to move the cursor from the rest position (origin of the axes) to a random target at a distance (D) and width (W) from the origin. Upward movement of the cursor represented open hand, downward movement represented closed hand, left represented wrist flexion while the right movement represented wrist extension. Based on the distance D and width W from the origin, each target’s index of difficulty (ID) was calculated. Various combinations of target distances and widths calculated by Equation (1) are tabulated in Table 3.

(1) $$ID=\log_{2}\left(\frac{D}{W}+1\right)$$

While testing in real-time, subjects were required to remain at a target for a dwell time of 1 s for the movement. Motion considered unsuccessful if the cursor remained in the target for less than one second [28,29]. Similarly, if the subject was unable to hit a target after 15 s of cue, the motion was considered unsuccessful and the cursor was moved back to the origin. To evaluate real-time system performance: path efficiency (PE), overshoot (OE), throughput (TP) and completion rate (CR) were examined as four performance parameters. Path efficiency (PE) is calculated by the distribution of the straight-line distance over the traveled distance [30]. It defines the quality of the control system. Overshoot is defined as the ability to stop at the target. It is the number of occurrences of the cursor being on the target and then leaving the target before the end of the 1s dwell time divided by the total number of targets [30]. Throughput (TP) is the ratio between the index of difficulty (ID) and the time taken (in seconds) to reach the target. The completion rate computes the percentage of successfully completed tasks within the time limit.

Offline classification performance parameters were computed using the obtained data. The training strategies were like the ones applied for online classification. Error_ij_ was defined as the ratio of misclassified decisions and total decisions. Between-day classification error (BCE) was computed by using the training data of the previous day and the testing data of the present day. Error_ij_ was calculated by Day i training data and Day j testing data. Within-day classification error (WCE) was computed by using training and testing data of the present day. Error_ij_ was computed by implementing two-fold validation. Combined-day classification error (CCE) was calculated using training data attained on all former days and present-day testing data.

### 2.5. Statistics

To evaluate the overall offline performance based on classification error, a nonparametric Friedman’s test with two-way layout with factor types (WCE, BCE and CCE) and the day (without Day 1) was used. *p*-values less than 0.05 were considered significant. To investigate the suitability of Fitts’ law test for the online experiment the relationship between completion time and index of difficulty was examined. The R^2^ coefficient of the linear model was examined to determine how the obtained data fit the computed linear model. For the overall performance based on each performance matric, nonparametric Friedman’s test was used to quantify the difference between days (without Day 1) and sessions. *p*-values less than 0.05 were considered significant. Results are presented as mean ± standard deviation.

## 3. Results

### 3.1. Offline Data Analysis

**Figure 3:**
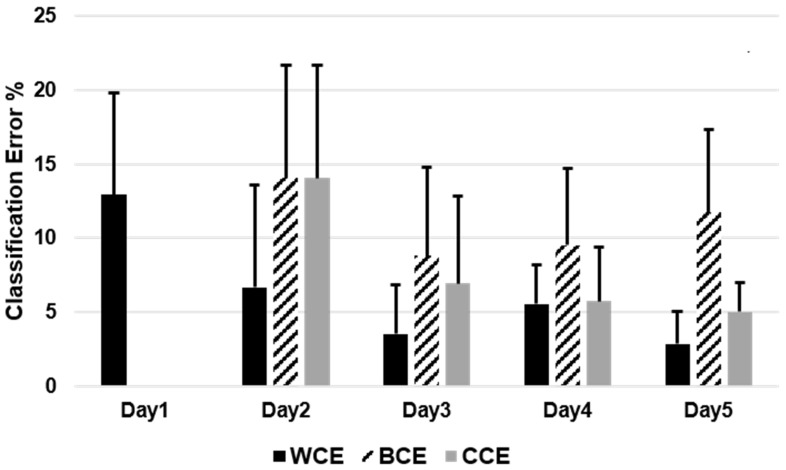
Comparison of percentage classification error (mean ± standard deviation) between each offline train/test scheme over five days.

Results showed that classification accuracies were significantly affected by training schemes (*p* ≤ 0.01) and but not with days (*p* = 0.09). Multiple comparison showed no significant difference (*p* = 0.41) between average WCE (4.6 ± 3.7%) and CCE (7.9 ± 4.8%). Classification performance improved over time in all train/test schemes but found no significance between days WCE (*p* = 0.06), CCE (*p* = 0.69), BCE (*p* = 0.18). The rate of improvement in classification performance was highest in WCE. It reduced to (2.6 ± 2.1%) on the final day from (12.9 ± 6.8%) on the first day. Figure 3 depicts the percentage classification error per training scheme in offline analysis.

### 3.2. Fitts’ Law Test (Online Results)

**Figure 4:**
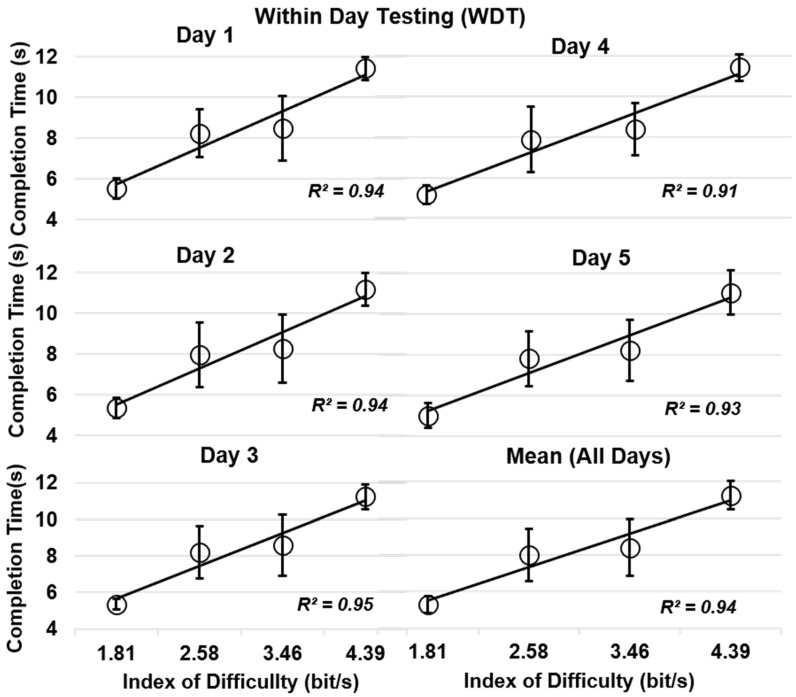
Relationship between completion time (CT; mean ± standard deviation) and index of difficulty for within-day testing (WDT).

The regression model depicted a strong linear relationship (coefficient of determination R^2^ ≥ 0.91) between completion time and index of difficulty in all three schemes. Figure 4 representing WDT was included in the paper. High values of correction between completion time and index of difficulty indicate the suitability of using the Fitts’ law test. By comparing training strategies with respect to IDs, it was found that CDT on average performed better than BDT (Table 4).

**Figure 5:**
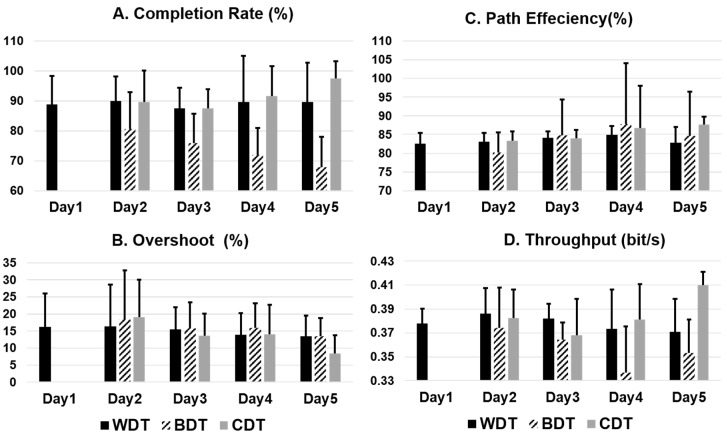
Comparison of real-time performance parameters over five days for all three testing schemes.

It was found that for BDT, completion rate reduced over days. It was lowest on Day 5 (Figure 5). Similarly, in CDT, completion rate improved over days. This has important implications in real-world conditions describing all the variations in the EMG signals over days that can be catered by including those variabilities in training data. Table 5 represents the summary of all performance parameters in three sessions averaged across all days.

**Figure 6:**
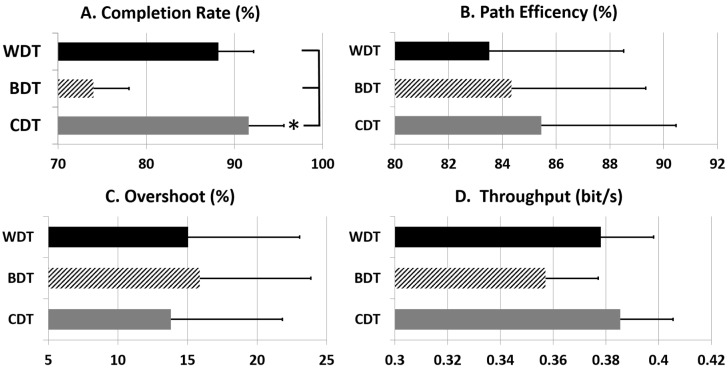
Comparison of performance parameters in all three testing schemes averaged across all days and sessions. Asterisks (*) indicate a case where there is a significant difference.

In Table 5, the results depict that for BDT, on average, the first session of each day was significantly better (*p* < 0.01) than the second and third sessions for completion rate (77.9 ± 14.0%) and path efficiency (88.9 ± 169%). Figure 6 represents the averaged values of all performance parameters across all days and sessions. Completion rate (CR; 91.6 ± 3.6%) of CDT was significantly better than BDT (74.0 ± 5.8%) and WDT (88.1 ± 3.6%). Overshoot (OS) was the lowest and path efficiency (PE) and throughput were the highest for CDT.

‘

## 4. Discussion

The use of iEMG has been the interest of scientists for many years. Providing the ability to control several prosthetic motions independently with greater dexterity, interday repeatability and intuitiveness is a laudable goal (as it outweighs surface EMG in these parameters). With the recent developments in the implantable sensor technology, these goals can be achieved. In this study, three strategies based on the training of a classifier were investigated over five days. The aim was to find the usability of iEMG in real-time and to find the effect of different training and testing strategies over multiple days in order to understand variabilities in EMG signals. This will help develop a robust myoelectric control and minimize the duration of recalibration. Fitts’ law test showed a high-value coefficient of determination (R^2^ > 0.91) for all days in all three investigated schemes.

Studies have shown that BCE increases as the time difference between training and testing of classifiers increases [23,31,32]. Offline results indicated a similar trend where a greater decrease in CCE was observed than BCE over days. Completion rate, one of the important performance parameters in the real-time tests, was found to be different between CDT, WDT and BDT over days as shown in Figure 5. Across all days, the completion rate (91.6 ± 3.6%) of CDT performed significantly better than BDT (74.0 ± 5.8%) and WDT (88.2 ± 3.6%). The trend of improvement in CDT and degradation in BDT represents the adaptation of the system. This effect can be more pronounced if the data can be combined for several days. Data from several days may also require deep networks to understand this process of adaptation of the system. The frequency of doing system recalibration of prostheses in a PR-based myoelectric control can be reduced if the deeper network is trained on long term data [31,32].

The completion time with respect to ID indicated differences in-between train/test strategies (Table 4). The performance metrics used in this study are comparable to intramuscular studies previously done [20,32]. Path efficiency describes the ability of the designed model to reach the target with the shortest distance. In this study, it was recorded (83.5 ± 4.4%), higher than reported in previous iEMG real-time studies (73.1% ± 2.8%, 77.09 ± 0.89%) [20,30]. Similarly, corresponding overshoot value was found low (15.0 ± 0.06%) compared to (22.1 ± 3.6%) in [30] and (56.3 ± 4.3%) in [20]. This explains the ability of the subject to hold at the target also improved over time.

Factors that can affect the surface-based real-time performance of myoelectric control are electrode shift, precipitation, skin impedance, interelectrode distances and electrode size. The overall trend in improvement could not be influenced by these factors in iEMG recordings. So, improvement in the real-time performance of WDT and CDT can only be described by the subject’s learning ability over time. This could improve further if the training time increases in the experiment. 

The quantification of outcomes is always a challenge with respect to arm function in myoelectric control studies. Most PR-based studies evaluated only classification accuracy as a performance metric. These offline results can further be validated by testing online. In this study, offline results reconciled with online tests, as offline analysis revealed that increasing training data over time, decreases the classification error. In a combined-day offline analysis (CCE), data trained on one day had a higher error (14.1 ± 7.6%) compared to data trained on four days concurrently (5.0 ± 2.1%). BCE and WCE analysis showed no significant difference in performance over days. Training strategies over a shorter duration of time had low errors, but they failed to encompass the normal variations in different hand motions. This problem can be solved by increasing the amount of training data for many days, to cater to natural variabilities in hand movements. However, unlike deep learning algorithms, the performance of classical machine learning algorithms degrades with large datasets that are spanned over days [32] and thus deep learning networks are encouraged to be used for future PR control schemes.

Challenging experimental protocol prohibited the acquisition of a sufficient number of subjects. However, results in this study illustrate a new research protocol and evaluation technique, which could be replicated in future case studies having more subjects and amputees. We believe that this study makes an important contribution to the literature.

## 5. Conclusions

In this study, we have developed a protocol to assess the real-time control parameters with fine-wire electrodes inserted in forearm muscles. We believe the protocol was a more challenging part that allowed recordings from inside muscles giving more insight to subchronic stability of performance parameters. A real-time evaluation of different training-testing schemes was demonstrated. Two DOF target acquisition tasks were tested using Fitts’ law. Both offline and online results suggest that time affects the robustness of real-time PR-based myoelectric control. Real-time performance can be enhanced by combining training data for many days (up to 100 days or more). Short-term experimental results may not represent the true performance of a control system, thus we recommend using training data of multiple days for better intuitive control of the prosthesis.

## Figures and Tables

**Table 1:** Complete scheme of the experiment. Three real-time tests were done on Days 2–5 and one on Day 1.

	Day 1	Day 2	Day 3	Day 4	Day 5
WDT	Train1 Test1	Train2 Test2	Train3 Test3	Train4 Test4	Train5 Test5
BDT		Train1 Test2	Train2 Test3	Train3 Test4	Train4 Test5
CDT		Train1–2 Test2	Train1–2–3 Test3	Train1–2–3–4 Test4	Train1–2–3–4–5 Test5

WDT - data from the same day. BDT - data collected from the previous day and tested on present-day.CDT - trained on all previous days including the present day and tested on present-day.

**Table 2:** Description of all features used in this study. N represents the total number of samples in a signal window, n is the sample index and ε is the threshold value.

Feature	Description	Formula
**MAV**	Mean Absolute Value (MAV) is the average of the absolute value of the EMG signal. It is an indication of muscle contraction levels.	$MAV=\;\frac{1}{N}\sum_{n=1}^{N}\left|x_{n}\right|$
**WL**	Waveform length (WL) is related to the fluctuations of a signal when the muscle is active. Thus, the feature provides combined information about the frequency, duration and waveform amplitude of the EMG signal.	$WL=\;\sum_{n=1}^{N-1}\left|x_{n}-x_{n+1}\right|$
**ZC**	Zero Crossing (ZC) measures the number of crosses by zero of the signal and is related to the frequency content of the signal. This feature provides an approximate estimation of frequency domain properties.	$ZC=\sum_{k=1}^{N-1}\left[\left(x_{n}\cdot x_{n+1}<0)\cap^{\text{​}}(\left|x_{n}-x_{n+1}\right|>\epsilon \right)\right]$
**SSC**	Slope Sign Changes (SSC) measures the number of times the sign changes in the slope of the signal. It is another method to represent the frequency information of the sEMG signal.	$SSC=\sum_{n=2}^{N-1}\left[\left(x_{n}-x_{n-1}\right)\cdot \left(x_{n}-x_{n+1}\right)\right]>\epsilon$
**WAMP**	Willison Amplitude (WAMP) estimates the number of active motor units, which is an indicator of the level of muscle contraction.	$WAMP=\sum_{n=1}^{N-1}\left|x_{n}-x_{n+1}\right|>\epsilon$
**CARD**	Cardinality of a set is a measure of the number of distinct values. This can be computed in two steps. Data needs to be sorted and one sample is distinct from the next if the difference is above a predefined threshold.	$\mathrm{Step}\;1:y_{n}=sort\left(x_{n}\right),\;n=1:N$ Step 2: $CARD=\sum_{n=1}^{N-1}\left|y_{n}-y_{n+1}\right|>\epsilon$

**Table 3:** Target distance (D) and width (W) from the origin. ID is the index of difficulty (in bits) of each target based on D and W.

Distance (D)	Width (W)	Index of Difficulty (ID)
**50**	5	3.46
**50**	10	2.59
**50**	20	1.81
**100**	5	4.39
**100**	10	3.46
**100**	20	2.59

**Table 4:** Comparison of completion time with respect to performance metrics for between-day testing (BDT), WDT, and combined-day testing (CDT).

	BDT	WDT	CDT
**1.81**	5.47 ± 1.45	5.31 ± 0.80	4.88 ± 0.56
**2.58**	8.45 ± 2.61	8.21 ± 2.74	8.04 ± 2.44
**3.46**	8.67 ± 2.78	8.63 ± 2.56	8.48 ± 2.48
**4.39**	11.71 ± 1.34	11.27 ± 0.87	10.97 ± 1.33

**Table 5:** Session wise difference in all three control schemes. Results depict averaged performance over five days per session. Asterisks (*) indicate a case where there is a significant difference.

Within-Day Testing (WDT)			
	***Session 1***	***Session 2***	***Session 3***
**Completion Rate**	90.3 ± 10.5	88.5 ± 10.2	88.7 ± 1.1
**Overshoot**	15.6 ± 8.5	14.5 ± 8.6	15.2 ± 9.1
**Path Efficiency**	83.4 ± 3.2	84.4 ± 3.3	82.7 ± 3.6
**Throughput**	38.1 ± 1.8	37.7 ± 2.6	37.6 ± 2.4
**Between-Day Testing (BDT)**			
	***Session1***	***Session 2***	***Session 3***
**Completion Rate**	77.9 ± 14.0 (*)	72.3 ± 15.9	71.9 ± 17.6
**Overshoot**	33.2 ± 10.8	33.5 ± 11.2	28.5 ± 5.8
**Path Efficiency**	88.9 ± 16.9 (*)	83.1 ± 9.1	81.1 ± 7.9
**Throughput**	35.8 ± 3.2	36.1 ± 3.2	35.1 ± 3.5
**Combined-Day Testing (CDT)**			
	***Session 1***	***Session 2***	***Session 3***
**Completion Rate**	94.0 ± 6.7	91.5 ± 9.5	89.4 ± 10.3
**Overshoot**	14.1 ± 11.0	13.0 ± 10.7	14.3 ± 11.6
**Path Efficiency**	85.6 ± 3.1	86.7 ± 3.6	84.1 ± 3.1
**Throughput**	39.2 ± 2.4	38.5 ± 2.9	38.0 ± 3.3
